# Inappropriate use of propranolol among medical and dental students at the University of Jordan: cross-sectional study

**DOI:** 10.3389/fmed.2025.1586068

**Published:** 2025-05-09

**Authors:** Hana Taha, Suhib Awamleh, AbdelRahman Al Tayyeb, Salwa Samhouri, Yousef Abbasi, Lujaien Alwaked, Aya El Jaber, Raseel Massad, Sireen M. Alkhaldi

**Affiliations:** ^1^Department of Family and Community Medicine, School of Medicine, University of Jordan, Amman, Jordan; ^2^Department of Neurobiology, Care Science and Society, Karolinska Institutet, Stockholm, Sweden

**Keywords:** beta-blockers, propranolol, medical students, dental students, self-medication, anxiety, misuse

## Abstract

**Aim:**

This paper aimed to investigate the prevalence of beta-blocker inappropriate use specifically propranolol, among medical and dental students at the University of Jordan. It examined the patterns of Propranolol consumption for stress management including frequency of usage, dosage, side effects experienced as well as the respondents’ level of awareness about the risks associated with unsupervised propranolol use.

**Methods:**

Cross-sectional study conducted at the University of Jordan in December 2024 and recruited 584 students (418 Medicine, 166 Dentistry). The data were analyzed using SPSS (version 27). Descriptive statistics were used to describe the sample, Pearson’s chi-squared test, fisher’s exact test, independent samples *t*-test and binary logistic regression model were used to identify the predictors of propranolol use. Statistical significance was set at a *p*-value ≤ 0.05.

**Results:**

Among the participants, 9.9% of the students reported using propranolol and 44% of the users learned about the medication through the recommendation of their friends and peers. Even though most students (74.1%) were aware of the potential risks of unsupervised propranolol use, still, 58.6% of the users took it without a prescription. The most common reasons for using propranolol were anxiety management (65.5%) and exam stress (60.3%). Most propranolol users (60.3%) noticed improvement in their academic performance and 36.2% of them experienced side effects such as dizziness and fatigue.

**Conclusion:**

Self-medication with propranolol among medical students to deal with academic anxiety carries serious risks. The findings of this research necessitate covering the potential hazards of self-prescription with beta-blockers within the curriculum of undergraduate medical and dental students. Moreover, there is a need for implementing student psychosocial support programs to improve their approach to managing stress and academic pressure.

## Introduction

Propranolol is a beta-adrenergic receptor antagonist that was developed more than 60 years ago (1). Due to its action on multiple receptor sites, propranolol is used in the treatment of many conditions including hypertension, cardiac arrhythmias, myocardial infarction, migraine, portal hypertension, hyperthyroidism, anxiety, essential tremors, and pheochromocytoma (2). Propranolol is a non-selective beta-blocker (BB) that blocks the action of catecholamines (adrenaline and noradrenaline) on beta-1 and beta-2 adrenergic receptors which inhibit the sympathetic effects that are activated by those receptors (3). This results in slowed heart rate, decreased oxygen demand, vasodilation, and reduced blood pressure (4, 5). Certain BBs, such as propranolol are lipophilic, enabling them to cross the blood-brain barrier and exert their effects on the Central Nervous System (CNS) (6). Through this extended spectrum of activity, it is evident that certain BBs can exhibit anxiolytic effects, which dampen the fight-or-flight response (7).

While propranolol may be effective in alleviating anxiety-related symptoms, it is frequently associated with side effects such as fatigue, insomnia, nightmares, dizziness, slow heart rate, and low blood pressure which can be exacerbated in the absence of proper medical supervision (5). Furthermore, propranolol is associated with potential neuropsychiatric complications (6). Propranolol is also known to induce depression type of organic mood disorder characterized by fatigue, weakness, and psychomotor retardation (8). Additionally, lipophilic BBs such as propranolol have been reported to disturb sleep continuity. Though the precise mechanism remains unclear, the study hypothesized it was through attachment to serotonergic receptors, crucial factors in the physiology of normal sleep (9). This particular side effect may undermine propranolol’s ability to reduce stress, as poor sleep is strongly correlated with heightened stress levels (10).

Propranolol may affect the metabolism of some medications such as antipyrine, chlorpromazine, theophylline and thyroid hormones (11). The decrease of plasma Triiodothyronine (T3), could be responsible for some of the metabolic responses to propranolol (12). A change in diet from high carbohydrates to high protein may result in increased oral clearance (13). Propranolol should be used with extreme caution in patients with diabetes because it might mask hypoglycemia symptoms including flushing, tachycardia, sweating, and dizziness (5). Moreover, dosages need to be adjusted to avoid toxicity in case of hepatic or renal insufficiency (5). Misuse of BBs can lead to serious adverse events and overdose can lead to severe cardiac arrest (14). Sudden withdrawal of propranolol should be avoided because it can lead to psychosis (15, 16) and life-threatening rebound angina, arrhythmias and infarction (17, 18). Ingestion of more than 1 g of propranolol in 24 h could be fatal and could lead to lethal bradycardia, bradyarrhythmia, hypotension, bronchospasm (5).

The ethical and legal implications of using propranolol in academic settings for performance anxiety are overlooked and understudied (19). Although propranolol is not approved by the American Food and Drug Administration (FDA) as anxiolytic, still it is procured in many countries over the counter (OTC), probably because it is perceived as a medication without abuse potential (19, 20). BBs are often used off-label by students experiencing exams-related stress (21). Butt et al., found that students who take BBs before exams had a significantly higher risk of taking antidepressants and a higher rate of suicide in the future (21). Thus, propranolol self-medication can be a warning sign about the more vulnerable students with psychological issues who need care.

Previous literature from Saudi Arabia have explored the prevalence of BBs inappropriate use among medical and dental students (22–27). A study conducted among Saudi medical and dental students found 30% use of propranolol (Inderal^®^) among the participants. Females and junior students from both specialties were less likely to use propranolol, whereas medical students were more likely to be propranolol users (24). Another study conducted by Abukhalaf et.al., in King Saud University found 22.4% prevalence of using BBs and 13.9% self-medication with BBs among medical and dental students. The most common reason for using BBs was to relieve stress and anxiety (25). A cross-sectional study was conducted at the College of Medicine, Taibah University, Al-Madinah Al-Munawwarah, Saudi Arabia found that 9.3% of the medical students used BBs during final exams and 76% of the BBs users were self-medicated without medical supervision (27).

A study conducted at the University of Jordan by Toubasi et al., found that 66.3% of medical students felt stressed (10). Another study conducted in Jordan by Masri et al., showed that medical students suffer from a high level of exhaustion (91%), disengagement (87%) and “minor” psychiatric illness (92%) (28). However, there is no research about the inappropriate use of Propranolol among medical students in Jordan. Thus, this is the first study that aims to investigate the prevalence of inappropriate use of BBs, specifically propranolol, among medical and dentistry students at the University of Jordan. It will also examine the patterns of propranolol consumption for stress management within this population, as well as assess the respondents’ level of knowledge regarding the risks associated with unsupervised propranolol use.

## Materials and methods

### Study design

This is a quantitative Cross-sectional study conducted in the School of Medicine at the University of Jordan from November 4 to December 14, 2024. The data was collected using voluntary anonymous self-administered online structured questionnaire.

### Study setting

Medical education in Jordan has developed significantly in recent decades. There are currently 8 medical schools in Jordan (2 private and 6 public) (29). The estimated total number of medical students in Jordan is 10,000, with approximately 1,500 graduates per year (29). The School of Medicine at the University Jordan was established in 1971. It is the first, the most prestigious and the top ranked college of medicine in the kingdom (30). There are 5,200 students enrolled with 69% females and 31% males (30). The academic year distribution includes 7.69% in the first year, 12.50% in the second year, 25.96% in the third year, 15.38% in the fourth year, 23.07% in the fifth year, and 15.38% in the sixth year. This distribution was considered when evaluating the representativeness of the sample.

### Measurement tool

A structured questionnaire was constructed based on pre-validated questionnaires from existing literature (24–26). However, the final set of questions were formulated by the authors and to ensure its relevance for our study population, the questionnaire underwent validation through content and face validity (31). The process involved the feedback of three experts in the fields of epidemiology and pharmacology. A pilot study was conducted with a sample size of *N* (138) to further reaffirm the relevance and validity of the questionnaire. The questionnaire consisted of 34 questions divided into five main segments. The sections included the following:

The first section captured sociodemographic characteristics, behavioral determinants of managing stress and past medical history. Sociodemographic information included: Age, gender, place of residence, year and program of study, weight and height. The behavioral determinants outlined the patterns of exercise, caffeine consumption, smoking and hours of sleep before examination. The second section focused on the usage of propranolol and how the participants firstly heard about propranolol and whether they were taking propranolol under a medical prescription. The third section further elaborated on the patterns of usage, frequency of usage, primary reason behind using propranolol and a question to indicate if participants notice academic improvement when using propranolol. The fourth section illustrated the doses of propranolol used and the approach of consuming propranolol before a stressful event. The final section assed the experienced side effects of propranolol and the extent of awareness regarding the side effects.

### Sample size and data collection

Convenience sampling was used as an appropriate method of recruiting participants due to feasibility and practicality of reaching the targeted population, as well as cost-effectiveness. Convenience sampling might impose certain limitation; however, this method was deemed suitable to answer the research question and any potential drawbacks are discussed in the limitations section.

The estimated sample size needed for this study was 357 participant and this was calculated using Cochran’s formula *N* = (Z^2^ p q)/e^2^ where, *N* is the sample size, *Z* is the *Z*-score statistic for 95% level of confidence which is 1.96, *P* is the expected prevalence which was estimated to be 18.06% based on approximating the mean prevalence from previous studies conducted in Saudia Arabia with similar populations (22–26, 32) and the margin of error = 0.05 (33). However, we recruited in this study a total of 584 students to ensure broader representation.

The questionnaire was placed on a Google form and was disseminated online through medical and dental students’ Facebook and WhatsApp groups. The inclusion criteria in this study were full-time undergraduate medical students from years 1-6 and dental students from years 1 to 5 at the University of Jordan aged 18–26. A total of 591 responses were recorded for the online questionnaire. However, 5 responses were excluded due to the refusal of consent to participation. Additionally, 2 responses presented with irreconcilable data were also excluded. These exclusions were made when responses were incomplete to the extent that essential variables required for analysis were missing or when contradictory information was provided, making it impossible to interpret or validate the data. The final data set was composed of *N* (584) students (418 Medicine, 166 Dentistry).

### Data analysis

Statistical Package for the Social Sciences (SPSS) version 27 was used to conduct the data analysis. The data collected in this research is discrete in nature, specifically focusing on the prevalence and perception of propranolol. To compare categorical variables between groups, Pearson’s Chi-squared tests were used. Fisher’s Exact Test (2-sided) was applied in cases where the expected frequency in any cell was less than 5 (specifically for smoking hookah status) and for “Knowledge of propranolol effect on stress and anxiety,” due to highly skewed distribution despite sufficient expected counts. Independent samples *t*-test was performed to investigate the correlation between a continuous variable and a binary variable. For GPA-related analyses, first-year students were excluded as their GPA data was unavailable. However, first-year students were included in all other analyses to ensure a comprehensive representation of academic years. A multivariate analysis was conducted using binary logistic regression model to identify predictors while accounting for potential confounders. The analysis included variables that were selected based on their significance at the bivariate level, as well as variables that were considered clinically or theoretically relevant based on previous research (22–26, 32). Additionally, variables identified as potential confounders, based on their established association with both the outcome and exposure regardless of bivariate significance. Results were reported as odds ratios (ORs) with 95% confidence intervals (CIs). Statistical significance was set at a *p*-value ≤ 0.05.

To check the internal reliability, we conducted Cronbach’s Alpha for a single-factor scale. The analysis yielded an Alpha value of 0.816, indicating good internal consistency. Since the questionnaire items used different response scales, the Standardized Cronbach’s Alpha was also measured, which came back as 0.946, indicating excellent reliability.

### Ethical considerations

Ethical approval to conduct the study was obtained from the University of Jordan Institutional Review Board (IRB) (Approval number 19/2024/781). The research adhered to strict ethical guidelines, with all participants giving their consent prior to their involvement in the study. Collected data was anonymized protecting participants identities.

## Results

### Baseline characteristics

The baseline characteristics of the 584 study participants were analyzed and demonstrated in Table 1. The mean age of the participants was 21.21. More than half of the participants were females (66%).

**TABLE 1:** Baseline characteristics.

Variable	Category	*N*	%
Age	Mean (± SD)	21.21	(± 1.592)
Gender	Male	199	34
	Female	385	66
Program	Medicine	418	71.6
	Dentistry	166	28.4
Academic level	First year	25	4.3
	Second year	68	11.6
	Third year	155	26.5
	Fourth year	88	15.1
	Fifth year	146	25.0
	Sixth year	102	17.5
Smoking (cigarettes	Yes	43	7.4
	No	541	92.6
	Mean (for cigarettes per day) (± SD)	10.47	(± 7.742)
Smoking (hookah)	Yes	35	6.0
	No	549	94.0
Smoking (vape or e-cigs)	Yes	58	9.9
	No	526	90.1
GPA	Mean (± SD)	3.39	(± 0.406)
Chronic diseases	None	532	91.1
	Migraine	25	4.3
	Other	27	4.6
Propranolol use	Yes	58	9.9
	No	526	90.1

The highest proportion of participants were in their third year (26.5%), followed by 25.0% in their fifth year. The mean GPA score was found to be 3.39/4.00, excluding the first-year students as at the time of the study, they still didn’t receive their GPA. The most common chronic disease affecting the participants was found to be migraine (4.3%). Approximately 10% (*n* = 58) of the participants were propranolol users and none of them were in their first year.

### Factors associated with propranolol use

Academic level, use of vapes/e-cigarettes, coffee and energy drinks consumption, and the knowledge of propranolol’s effect on stress and anxiety were all found to be significantly associated with propranolol use among the study participants. Table 2 shows the results of different chi-square tests that were conducted to assess the different variables associated with the use of propranolol.

**TABLE 2:** Factors associated with propranolol off-label use.

	Have you ever used propranolol?						
**Variable**	**Categories**	**No**		**Yes**		**χ^2^**	***p*-value**
		***N***	**%**	***N***	**%**		
Gender	Female	347	66	38	65.5	0.006	0.938
	Male	179	34	20	34.5		
Program	Medicine	372	70.7	46	79.3	1.939	0.164
	Dentistry	154	29.3	12	20.7		
Academic level	Pre-clinical years	231	43.	17	29.3	4.432	0.035
	Clinical years	295	56.1	41	70.7		
Smoking (cigarettes)	Yes	35	6.7	8	13.8	4.161	0.041
	No	491	93.3	50	86.2		
Smoking (hookah)	Yes	32	6.1	3	5.2	–	1.000^a^
	No	494	93.9	55	94.8		
Smoking (vape/e-cigarettes)	Yes	48	9.1	10	17.2	3.801	0.051
	No	478	90.9	48	82.8		
Coffee/energy drinks consumption	Yes	345	65.6	47	81.0	5.731	0.017
	No	181	34.4	11	19.0		
Knowledge of propranolol effect on stress and anxiety	Yes	322	61.2	54	93.1	–	<0.001^a^
	No	202	38.8	4	6.9		

^a^Fisher’s Exact Test (2-sided) used. For “Smoking (hookah)”, due to low expected cell count; for “Knowledge of propranolol effect on stress and anxiety”, due to highly skewed distribution despite sufficient expected counts.

A multivariate analysis using binary logistic regression was conducted alongside the Hosmer-Lemeshow test to evaluate the goodness-of-fit of the model used, and it yielded a p-value of 0.127, indicating that the model provides adequate fit to the data. Results of the multivariate analysis are in Table 3. We found that cigarette smoking, and coffee consumption were both significantly associated with propranolol use on a bivariate level; but when accounting for other predictors, they were not significant. An independent sample *t*-test revealed that there was no statistically significant relationship between the average hours slept before an exam and propranolol use [*p*-value = 0.136 (95% CI = −0.840–0.115)].

**TABLE 3:** Multivariate analysis of propranolol use predictors.

Predictor	Odds ratio	95% CI	*p*-value
Gender	1.151	0.619–2.142	0.657
Program	0.865	0.430–1.740	0.684
Academic level (pre *vs.* clinical)	1.065	0.563–2.016	0.847
Smoking cigarettes	0.480	0.195–1.185	0.111
Coffee/energy drinks consumption	0.497	0.246–1.003	0.051
Knowledge of propranolol effect on stress and anxiety	0.128	0.043–0.375	<0.001
GPA	0.978	0.693–1.382	0.901

### Propranolol use patterns

Propranolol users for the most part (44.8%) learned about propranolol through the recommendation of their friends and peers. Around 29.3% reported using it only pre-examinations, with the most common dose being 10 mg (72.4%). Most participants (60.3%) noticed improvements in their academic performance after using propranolol. Table 4 shows propranolol use pattern, and it demonstrates no statistically significant relationship (*p* = 0.94) between experiencing side effects and the likelihood of recommending propranolol to others.

**TABLE 4:** Propranolol use pattern.

Question	Category	*N*	%
How did you first learn about propranolol?	Doctor’s prescription	24	41.4
	Friend/peer recommendation	26	44.8
	Internet research	5	8.6
	Medical school curriculum	3	5.2
How frequently do you use propranolol?	Frequently (a few times a month)	5	8.6
	Occasionally (once a month or less)	14	24.1
	Once	16	27.6
	Only pre-examinations	17	29.3
	Regularly (weekly or more)	6	10.3
Have you noticed any improvements in academic performance when using propranolol?	Yes	35	60.3
	No	23	39.7
Was propranolol prescribed to you by a health care professional?	Yes	24	41.4
	No	34	58.6
Dosage	5 mg	1	1.7
	10 mg	42	72.4
	20 mg	7	12.1
	40 mg	7	12.1
	Missing data	1	1.7
How effective do you find propranolol in managing your symptoms?	Extremely effective	6	10.3
	Very effective	21	36.2
	Effective	21	36.2
	Somewhat effective	8	13.8
	Not effective at all	2	3.4
How long before a stressful event do you usually take propranolol?	Less than 30 min	14	24.1
	30–60 min	25	43.1
	1–2 h	16	27.6
	More than 2 h	3	5.2
Have you ever experienced any side effects from propranolol?	Yes	21	36.2
	No	37	63.8
Are you aware of the potential risks of long-term use of propranolol?	Yes	43	74.1
	No	15	25.9
Would you recommend propranolol use to others?	Yes	28	48.3
	No	30	51.7
Do you feel adequately informed about propranolol and its effects?	Yes	39	67.2
	No	19	32.8
What is the primary reason for your propranolol use?^a^	Exam stress	35	60.3
	Anxiety management	38	65.5
	Public speaking	13	22.4
	Palpitations	3	5.2
	Other	5	8.6
What side effects have you experienced?^a^	Dizziness	11	50
	Nausea	3	13.6
	Cold hands	1	4.5
	Fatigue	11	50
	Bradycardia	8	36.4
	Sleep problems	1	4.5

^a^Multiple answers were selected, percentage refers to percent of cases.

**FIGURE 1:**
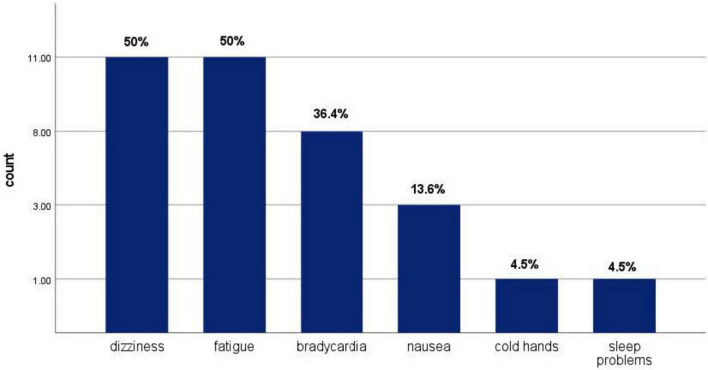
Side effects experienced by propranolol user.

**FIGURE 2:**
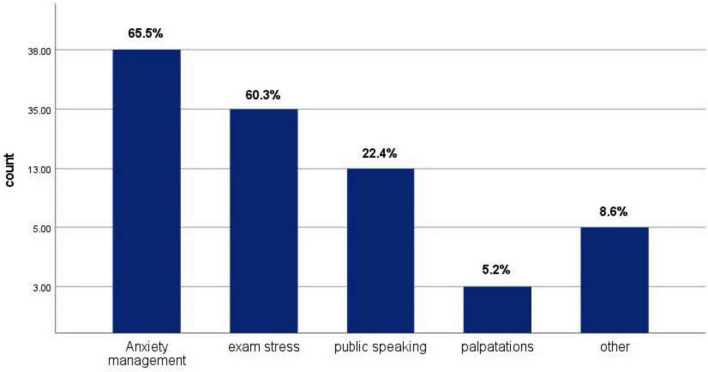
Primary reported reasons for propranolol user.

Figure 1 shows the most common side effects experienced by propranolol users, with dizziness and fatigue being the two most common side effects, each affecting 50% of users who experienced side effects, followed by bradycardia (36.4%) as the second most common side effect. Figure 2 shows the primary reported reasons for propranolol use, with the most common being anxiety management (65.5%), followed by exam stress (60.3%) and public speaking (22.4%).

## Discussion

This cross-sectional study was conducted at the University of Jordan to investigate the prevalence of propranolol use among students enrolled in the schools of Medicine and Dentistry. There is no prior research from Jordan about this research topic. Thus, our study builds on prior research which primarily took place in other Middle Eastern universities, exploring similar demographics (3, 22–26, 32, 34, 35). Propranolol has emerged as a significant pharmaceutical agent, primarily intended for the control of cardiac-related disorders. Beyond the primary use of the drug, certain BBs are lipophilic and can cross the blood-brain barrier (6). This mechanism of action renders it effective in relieving anxiety symptoms.

Our study revealed a relatively low adoption of propranolol among medical and dentistry students (9.9%) which contrasts with the findings by Al-Mohrej et al. at a university in Jeddah, Saudi Arabia, which reported a higher adoption rate of 30%. However, our results align with the findings of Alkhatabi et al. (26) which conducted a study in King Saud Bin Abdul Aziz University for Health Sciences and found the prevalence of propranolol to be 14.4% among medical students. Our findings are also consistent with a study conducted by Aljahdali et al., among health sciences students at Umm Al-Qura University in Makkah, Saudi Arabia that found 6.5% off-label use of propranolol (22). Another cross-sectional study conducted by Tounsi et al., in Riyadh dental schools found that nearly 12% of dental students used propranolol for non-medical reasons (32).

Our results showed that demographics such as gender and study program were not significantly associated with the use of propranolol, according to our yielded *p*-values of 0.938 and 0.164, respectively. Even after multivariate analysis and taking confounding variables into account, the predictors remained insignificant. This is consistent with the findings of a previous study by Altalhi et al. (23) that targeted medical students in all the districts of Saudi Arabia.

Our findings indicated that propranolol use was most prevalent among students in their clinical years, while none of the first-year students reported using the drug. This aligns with the research results of Abukhalaf et al. (25), Altalhi et al. (23), and Alkhatabi et al. (26) which showed an increase in propranolol use during the clinical years of undergraduate medical education. The results of previous studies showed a striking correlation between off-label use of propranolol with the stress associated to Objectively Structured Clinical Examination (OSCE) (22–26). According to a previous study on medical students’ views of various examinations and assessment methods, 63% of participants indicated that OSCE was extremely stressful for them, and 50% said the assigned time for OSCE gave them short period for studying (36). The most conspicuous uses for propranolol in Alkhatabi et al., study revolved around exam anxiety relief and performance enhancement for OSCEs (70.6%), and before oral presentations (38.2%) (26). Similar results were reported by Al-Mohrej et al. (24) who found anxiety reduction to be the most common reason for use. This effectiveness in alleviating anxiety symptoms is related to the aforementioned ability of propranolol to exert its effect on the CNS (8). However, this also carries the risk of adverse effects that span from dizziness, fatigue, bradycardia to life-threatening rebound angina, arrhythmias and infarction (5).

Almost half of the propranolol users in our study recommended the medication to other students, independently of experiencing side effects. This behavior may be attributed to medical students’ tendency to self-medicate, as highlighted by Pandya et al., where 82.3% of medical students reported self-medication within the last year (37). This is also consistent with the results of a study conducted by El Ezz et al. at Ain Shams University, Egypt, that found 55% prevalence of self-medication among medical students. From these, 60% increased the dose without medical advice and 4.8% reported side effects (35). Similarly, our study found that 58.6% of the propranolol users took it without a prescription from a healthcare professional. This could be explained by the fact that most students (93.1%) in our study were aware of propranolol’s effect on anxiety and stress. This made them more likely to use the medication as evidenced by a statistically significant *p*-value of < 0.001, which remained significant after conducting a multivariate analysis that accounted for confounding variables such as program and academic level. A study by Pandya et al. (37) found a significant relationship between the level of education and heightened exam stress with the increased the likelihood of self-medication. The most prevalent reasons for this were documented as timesaving and convenience, which are crucial considerations in the lives of medical and dentistry students.

Although our study found no significant relationship between GPA and propranolol use (*p*-value 0.901), most students (60.3%) reported feeling like propranolol enhanced their academic performance. Propranolol is significantly associated with improved examination performance including mental arithmetic and verbal reasoning scores, especially among students with anxiety (38). A National Survey of 3,326 medical and pharmacy students in Saudi Arabia found that students who are taking BBs are those at high risk of underlying anxiety disorders (34). This can be attributed to propranolol’s efficacy in providing relief to students in stress-induced environments like examinations (7). However, it is essential to acknowledge the possible placebo effect in the perceived effectiveness of BBs in managing stress among students. Despite a substantial portion of our population reporting that they felt propranolol was effective in alleviating their anxiety symptoms, the systematic review by Archer et al., shows a lack of tangible evidence that supports the effectiveness of BBs in managing anxiety (39). The absence of definitive efficacy suggests that the reported improvement by some users could potentially be influenced by their expectations of the anxiolytic effects rather than direct pharmacological effects (39). This is also supported by our findings that showed no statistically significant association between GPA and propranolol use.

### Limitations

Not all the factors that could be associated with propranolol misuse were addressed in this study. The primary focus was on sociodemographic aspects, while psychological or clinical motivations behind the students’ decisions to use propranolol were not explored. Binary outcomes simplified the data analysis and allowed for the application of logistic regression, chi-square tests, and binary classification algorithms. This provided valuable insights and enhanced the interpretability of the results. However, there is potential for imbalanced results and biased predictions. Our findings should be interpreted within the context of a number of limitations. Firstly, we used convenience sampling, and all the participants were from the University of Jordan. This may impact the generalizability of our results to the broader population of Jordanian medical students. Secondly, the study’s cross-sectional design is inherently associated with a number of drawbacks including snap-shot prevalence results and inability to establish temporal relationships or causality. Thirdly, the data was collected using an online self-administered questionnaire. Despite its validation from the literature, self-reported measures are subject to social desirability bias, recall bias, or neutral bias. Finally, we acknowledge that Hosmer-Lemeshow goodness-of-fit weaknesses of limited power and poor interpretability (40, 41).

### Recommendations

To improve the quality of future studies it is recommended to use a randomized sampling method to reduce bias. Additionally, upcoming research should be conducted in several private and public medical schools to allow for better comparisons and critical assessment of the findings to enhance generalizability and improve validity. Moreover, the issue of propranolol use without adequate medical supervision needs to be addressed. Our study reported that students, despite being aware of the side effects, continue to use as well as recommend propranolol to others. This raises concerns regarding the potential long-term side effects of the drug. Students must be educated on the dangers of self-medication regardless of their level of medical knowledge. Therefore, we recommend that educational initiatives be placed to educate students on the harmful effects of self-medication. Universities should also offer anxiety-management courses to help students find healthier ways to cope with academic-induced anxiety.

## Conclusion

In this study, the prevalence of propranolol use was found to be 9.9%. Despite the high level of awareness about the possible side effects of propranolol, self-medication with propranolol by medical and dental students was prevalent. Among the students using propranolol, 58.6% took the medication without any supervision. Self-medication for the management of anxiety-related symptoms was evident, and it should not be taken lightly, especially when dealing with medications such as propranolol. The causal associations between potential sociodemographic and academic determinants with the inappropriate use of propranolol should be addressed through longitudinal studies. There is also a need for qualitative research to better understand the underlying reasons behind the inappropriate use of propranolol. This is necessary for designing context sensitive programs to address the problem of self-medication and to improve students’ management of anxiety and academic pressure.

## Data Availability

The raw data supporting the conclusions of this article will be made available by the authors, without undue reservation.
